# Effect of anti-interleukin-5 antibody on development of vasculitis in an ovalbumin-induced eosinophilic vasculitis mouse model

**DOI:** 10.3389/fphar.2025.1546785

**Published:** 2025-03-19

**Authors:** Kiyoto Kageyama, Eri Kikuchi, Nao Hoshino

**Affiliations:** Discovery Research Laboratories, Nippon Shinyaku Co., Ltd., Kyoto, Japan

**Keywords:** EGPA, IL-5, ovalbumin, eosinophilic vasculitis, mouse model

## Abstract

**Background:**

Eosinophilic granulomatosis with polyangiitis (EGPA) is a rare and intractable chronic disease. Glucocorticoids are the mainstay of treatment for EGPA. The drugs that target interleukin (IL)-5 signaling have also been marketed or developed in recent years. While no animal model can completely recapitulate EGPA, the ovalbumin (OVA)-induced eosinophilic vasculitis mouse model exhibits pathological similarities in the lungs. However, the effect of an anti-IL-5 drug has not yet been investigated using this model. This study used the OVA-induced eosinophilic vasculitis model to evaluate and characterize its usefulness, focusing on the effects of an anti-IL-5 antibody.

**Methods:**

Female C57BL/6NCrSlc mice were intraperitoneally immunized on days 0 and 14 with OVA adsorbed onto an aluminum gel. From days 26–32, the mice were exposed to aerosolized 1% OVA daily for 1 h. Anti-IL-5 antibody or vehicle was injected intravenously or intraperitoneally once on the first day of aerosol exposure (day 26). On day 33, the eosinophil and lymphocyte counts in the blood and bronchoalveolar lavage fluid (BALF) were measured. Lung specimens were used to assess the vascular lesions formed in the pulmonary arteries. Plasma cytokine levels were measured on day 28.

**Results:**

The anti-IL-5 antibody significantly reduced eosinophil counts in the blood and BALF. In contrast, it did not inhibit the lymphocyte counts in BALF or vascular lesion formation. The anti-IL-5 antibody significantly blocked the plasma level of IL-5 on day 28. However, the levels of other cytokines (i.e., IL-2, IL-6, tumor necrosis factor-α, granulocyte-macrophage colony-stimulating factor, interferon-γ, IL-12, IL-4, IL-13, and IL-17) were not altered.

**Conclusion:**

In the investigated model, lymphocyte infiltration in lung tissue and cytokines other than or in addition to IL-5 were suggested to have contributed to the development of vasculitis. IL-5 signaling has a potential impact on EGPA pathogenesis via a different mechanism or in addition to the mechanism demonstrated in the OVA-induced eosinophilic vasculitis mouse model. The model may be useful for drug discovery targeting both eosinophilic and non-eosinophilic aspects of EGPA pathogenesis.

## 1 Introduction

Eosinophilic granulomatosis with polyangiitis (EGPA) is an antineutrophil cytoplasmic antibody (ANCA)-associated vasculitis, distinct from other major forms of ANCA-associated vasculitis, such as granulomatosis with polyangiitis and microscopic polyangiitis. EGPA is a rare and intractable chronic disease characterized by a marked increase in peripheral blood eosinophil counts, often occurring against a background of allergic diseases like bronchial asthma. Its pathophysiological characteristics generally include eosinophilic inflammation in systemic organs and granulomatous necrotizing vasculitis in small-to-medium-sized blood vessels. The eosinophilic inflammation and ANCA-mediated inflammation involving immune cells other than eosinophils contribute to the development of EGPA pathology. The clinical symptoms and progression of the disease may vary depending on genetic background and environmental factors. ANCA-negative patients are genetically closer to asthma and tend to frequently exhibit heart failure. On the other hand, ANCA-positive patients share genetic characteristics with other ANCA-associated vasculitis, and tend to frequently exhibit glomerulonephritis, alveolar hemorrhage, and neuropathy (Furuta et al., 2019). Furthermore, several cytokines are involved in the pathogenesis and disease activity of EGPA (Vaglio et al., 2013; Rodriguez-Pla et al., 2020).

Pharmacotherapy for EGPA includes high-dose glucocorticoids used to induce remission in combination with or without immunosuppressive drugs. With time, the dosage of glucocorticoids is gradually reduced (Chung et al., 2021; Hellmich et al., 2024). Mepolizumab (NUCALA^®^) is an antibody targeting interleukin (IL)-5, a cytokine that regulates eosinophils, which has been approved for the treatment of EGPA. Clinical trials have shown that mepolizumab reduces the required dose of glucocorticoids and suppresses EGPA flare-ups (Wechsler et al., 2017). Benralizumab (FASENRA^®^), an antibody against the IL-5 receptor α subunit (IL-5Rα) expressed mainly on eosinophils, was investigated in a phase 3 trial in comparison with mepolizumab. In addition to the inhibition of IL-5 signaling, benralizumab mediates the apoptosis of eosinophils via antibody-dependent cell-mediated cytotoxicity (Kolbeck et al., 2010). In a phase 3 trial, benralizumab was found to be comparable to mepolizumab with respect to achieving remission at weeks 36 and 48 (Wechsler et al., 2024); the incidence of adverse events and serious adverse events was comparable between both groups. However, a previous study has shown that patients with low eosinophil counts do not respond well to mepolizumab (Wechsler et al., 2017).

As the first animal model of eosinophilic vasculitis, an ovalbumin (OVA)-induced eosinophilic vasculitis mouse model was previously developed (Yamauchi et al., 2010). The study demonstrated a remarkable obstructive remodeling of small pulmonary arteries in OVA-sensitized mice subjected to repetitive OVA inhalation. In addition to obstructive changes, extravascular eosinophilic infiltration and granulomas were also observed. These pathological changes recapitulate the aspects of allergic granulomatous angiitis, and hence, this mouse model could contribute to the understanding of allergic granulomatous angiitis. The disease “allergic granulomatous angiitis” has now been renamed EGPA. This mouse model has been used to evaluate various drugs (e.g., imatinib, a platelet-derived growth factor and a receptor tyrosine kinase inhibitor; rapamycin, a mammalian target of rapamycin inhibitor; ZSTK474, a phosphatidylinositol 3-kinase inhibitor; and tofacitinib, a Janus kinase inhibitor) in other studies (Suzuki et al., 2013; Koizumi et al., 2014; Oikawa et al., 2016; Matsumoto et al., 2019).

To date, the effect of an anti-IL-5 antibody in this model has not been confirmed. Therefore, in this study, we aimed to evaluate the OVA-induced eosinophilic vasculitis model and characterize its usefulness in elucidating the effects of an anti-IL-5 antibody.

## 2 Materials and methods

### 2.1 Materials

The anti-IL-5 antibody reslizumab (CINQAIR^®^) was purchased from Teva Pharmaceutical Industries (Tel Aviv, Israel).

### 2.2 Animals

Female C57BL/6NCrSlc mice (5 weeks old) were purchased from a commercial breeder (Japan SLC, Inc., Shizuoka, Japan) and housed in the animal facilities of Nippon Shinyaku Co., Ltd. throughout the study under environmental control, including light exposure (lights on from 08:00 to 20:00 h), ventilation (more than 15 air changes per hour), temperature (23°C ± 3°C), and humidity (55% ± 20%). The mice were provided *ad libitum* access to pellet chow (Funabashi Farm, Funabashi, Japan) and tap water following quarantine and acclimation. This study was conducted in compliance with the Internal Regulations on Animal Experiments at Nippon Shinyaku Co., Ltd, which are based on the Law for the Humane Treatment and Management of Animals (Law No. 105, 1 October 1973, as amended on 19 June 2019). The Animal Experimental Ethics Committee approved the study protocol (approval number: C2020301).

### 2.3 Mouse model of OVA-induced eosinophilic vasculitis

**FIGURE 1:**
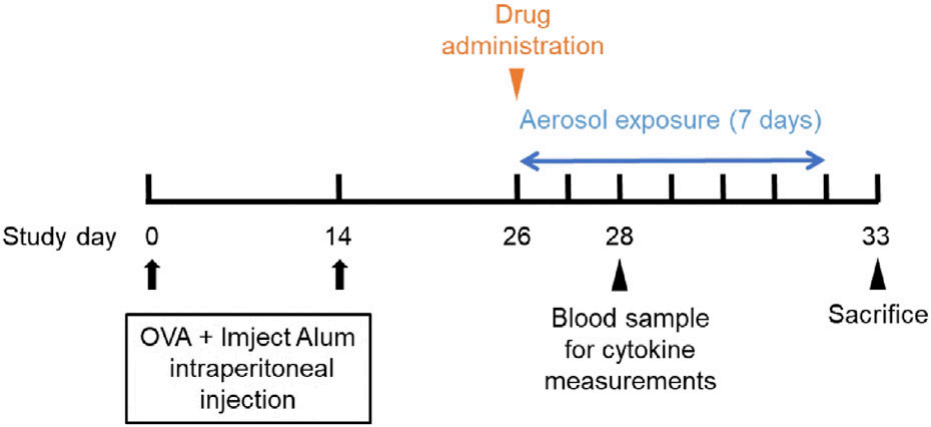
Schedule of the study.

**FIGURE 2:**
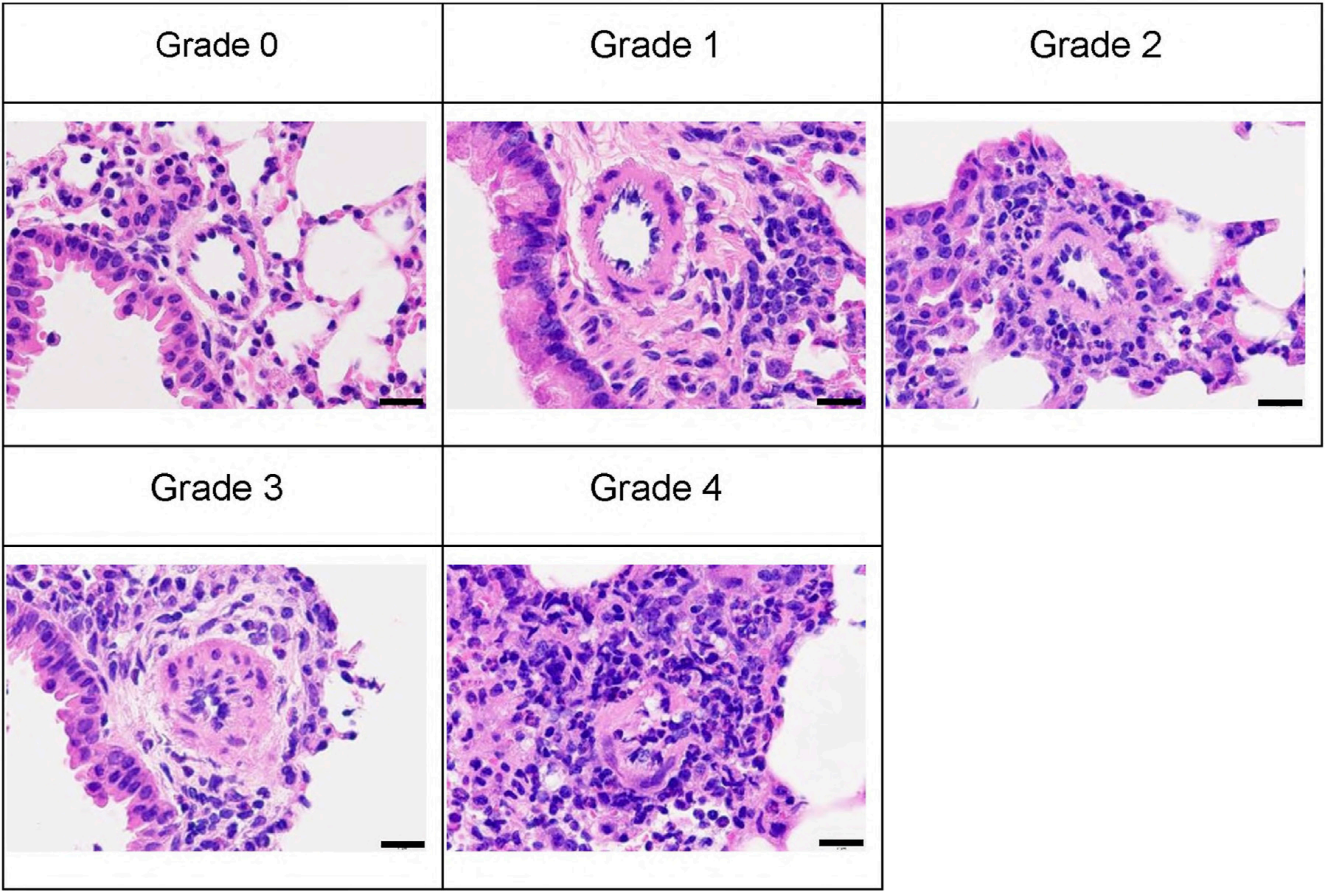
Representative images of each vascular lesion grade stained with hematoxylin and eosin; scale bars represent 20 µm. An analysis of ten arteries (diameter 40–70 μm) was graded as follows. Grades 0, no abnormality; 1, minimal (no change in the vascular smooth muscle layer and minimal perivascular inflammatory cell infiltration); 2, mild (no change in the vascular smooth muscle layer and mild perivascular inflammatory cell infiltration); 3, moderate (thickening of the vascular smooth muscle layer with/without inflammatory cell infiltration); 4, severe (artery injury such as disruption of internal elastic laminae and proliferation of mesenchymal cells in the intraluminal space in arteries). Group animal numbers (vessel numbers) for each grade of the representative pictures are as follows: Grade 0, saline/vehicle group-1 (9); grade 1, 1% OVA/anti-IL-5 antibody 25 mg/kg, i.v. group-4 (6); grade 2, 1% OVA/anti-IL-5 antibody 10 mg/kg, i.v. group-5 (5); grade 3, 1% OVA/vehicle group-1 (8); grade 4, 1% OVA/anti-IL-5 antibody 10 mg/kg, i.v. group-1 (8); i.v., intravenous.

The previously developed mouse model of OVA-induced eosinophilic vasculitis (Yamauchi et al., 2010) was optimized for this study (Figure 1). Briefly, the day of the first immunization was defined as day 0. Female C57BL/6NCrSlc mice (6 weeks old) were immunized intraperitoneally on days 0 and 14 with OVA (8 μg/mouse; Sigma-Aldrich, Steinheim, Germany) adsorbed onto 20% aluminum gel (Imject Alum, Thermo Fisher Scientific, Sunnyvale, CA, USA) in phosphate-buffered saline (PBS) (−). All mice, including those in the saline aerosolization group, were immunized. The mice were then divided into groups of five or seven by stratified randomization with body weights on day 25. Reslizumab or vehicle (saline) was injected intravenously (i.v.) or intraperitoneally (i.p.) once on the first day of aerosol exposure (day 26). From days 26–32, the mice were placed in a plastic chamber (23 × 23 × 27 cm, custom-made by Natsume Seisakusho, Tokyo, Japan) and exposed to aerosolized 1% OVA (weight/volume) saline solution for 1 h daily using a nebulizer (PARI BOY SX, Pari GmbH, Starnberg, Germany). The negative control group (saline/vehicle) was exposed to aerosolized saline and received intravenous saline. The positive control group (1% OVA/vehicle) was exposed to aerosolized 1% OVA and received intravenous saline. A total of 40 µL blood was drawn from the tail on the third day of inhalation (day 28), and plasma was obtained for cytokine measurements. The cytokine concentrations were measured using a Bio-Plex 200 system (Bio-Rad Laboratories, Hercules, CA, USA). On day 33, the mice were anesthetized with isoflurane, and blood was drawn from the heart. Blood samples were tested using a hematology system (ADVIA 2120i; Siemens Healthcare K.K., Tokyo, Japan), and the lymphocyte and eosinophil counts were measured. The bronchiole leading to the left upper lobe was tied with a string, and the trachea was cannulated using a tube. The lungs were lavaged with buffer [0.1% bovine serum albumin (weight/volume), and 50 μM ethylenediaminetetraacetic acid-2Na in PBS (−)]. The bronchoalveolar lavage fluid (BALF) was centrifuged to pellet the cells, the supernatant was removed, and the cell pellet was resuspended in 0.5 mL of the same buffer. Hematological analysis was then performed on this cell suspension using a hematology system (ADVIA 2120i). The lymphocyte and eosinophil counts were evaluated. The left upper lobe of the lung was fixed with 10% neutral-buffered formalin. Hematoxylin and eosin-stained specimens were analyzed to evaluate pathological changes in the pulmonary arteries. A total of 10 arteries (diameter 40–70 μm) were analyzed following the method outlined in the previous study (Yamauchi et al., 2010). Representative images of the vascular lesions within each grade are shown (Figure 2). The average grade of the ten arteries was used to represent the grade of each mouse.

### 2.4 Statistical analysis

Statistical analyses were performed using SAS 9.3 (SAS Institute, Cary, NC, USA). Eosinophil and lymphocyte counts in the blood and BALF, pulmonary artery pathological grade (vascular lesion grade), and blood cytokine levels were analyzed. First, a statistical comparison between the saline/vehicle (n = 5) and 1% OVA/vehicle (n = 7) groups was performed using Student’s t*-*test. When statistically significant, statistical comparisons of the 1% OVA/vehicle group and the treatment groups (n = 7) were performed using Dunnett’s multiple comparison test. *P* < 0.05 was considered statistically significant.

## 3 Results

### 3.1 Effects of anti-IL-5 antibody on eosinophil and lymphocyte counts and development of vasculitis in the eosinophilic vasculitis mouse model

The efficacy of the anti-IL-5 antibody in the mouse model of OVA-induced eosinophilic vasculitis was investigated.

**FIGURE 3:**
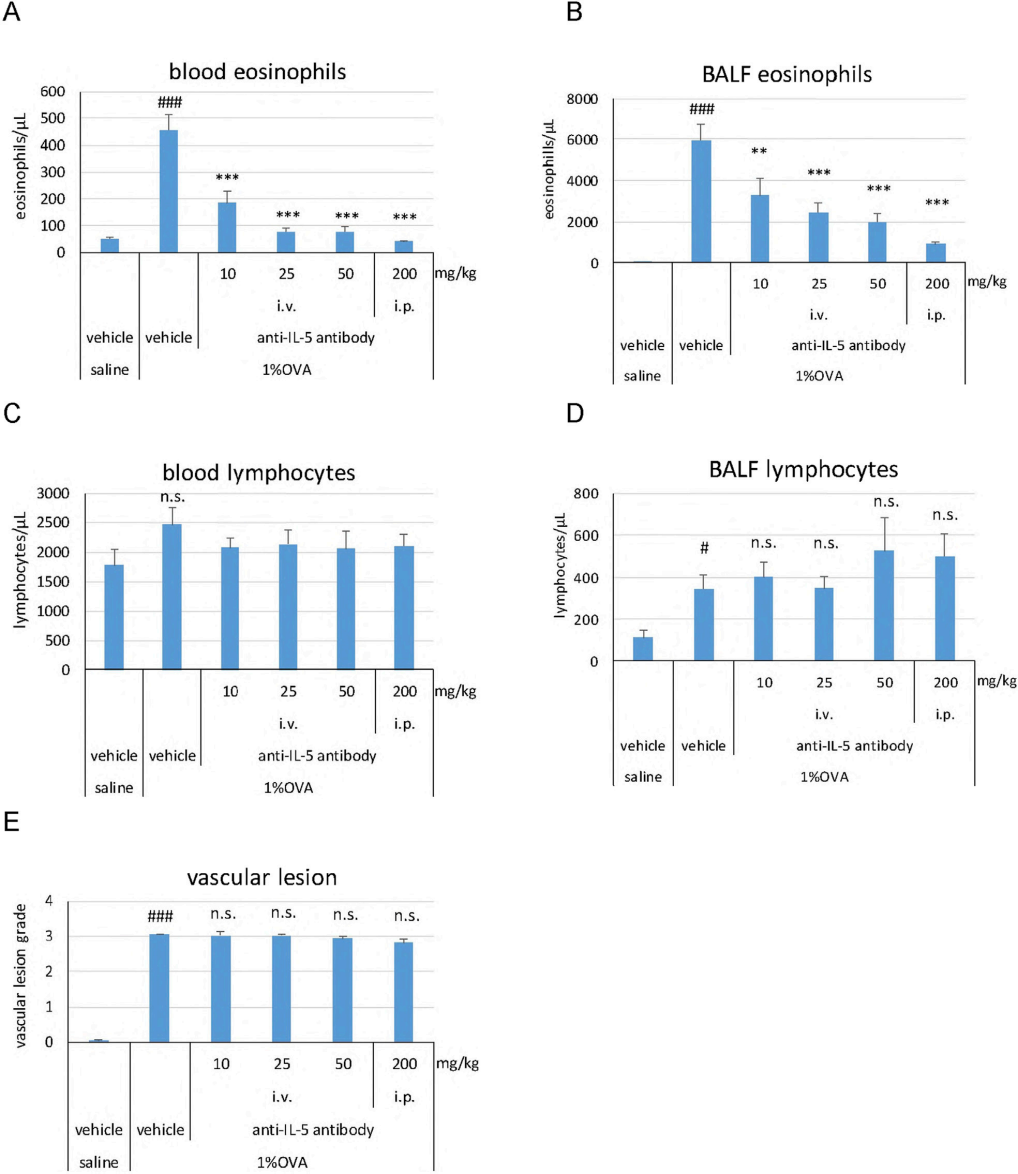
Effects of the anti-IL-5 antibody on eosinophils, lymphocytes, and vasculitis in the mouse eosinophilic vasculitis model. Blood eosinophil counts **(A)**; BALF eosinophil counts **(B)**; Blood lymphocyte counts **(C)**; BALF lymphocyte counts **(D)**; Histopathological changes in the pulmonary arteries **(E)**. i.v., intravenous; i.p., intraperitoneal. Data are expressed as the mean +SEM, n = 5–7. #*P* < 0.05, ###*P* < 0.001 vs. saline/vehicle group (Student's *t*-test), **P < 0.01, ***P < 0.001 vs. 1%OVA/vehicle group (Dunnett’s multiple comparison test). n.s., not significant.

Eosinophil counts in the blood (*P* < 0.001) and BALF (*P* < 0.001) were significantly higher in the 1% OVA/vehicle group than in the saline/vehicle group (Figures 3A, B). The anti-IL-5 antibody significantly and dose-dependently reduced the eosinophil counts in the blood (*P* < 0.001 at all doses) and BALF (*P* < 0.01 at 10 mg/kg, *P* < 0.001 at 25, 50, and 200 mg/kg) compared to that of the 1% OVA/vehicle group (Figures 3A, B). The anti-IL-5 antibody (200 mg/kg, i.p.) reduced the blood eosinophil counts to a level of that in the saline/vehicle group (Figure 3A). No clear changes were observed in the blood lymphocyte counts in any of the groups (Figure 3C). The lymphocyte counts in BALF were significantly higher (*P* < 0.05) in the 1% OVA/vehicle group than in the saline/vehicle group (Figure 3D). However, at any dose, the anti-IL-5 antibody did not reduce the lymphocyte counts in the BALF compared to that of the 1% OVA/vehicle group (Figure 3D). The development of vasculitis, represented by vascular lesion grade, was significantly higher (*P* < 0.001) in the 1% OVA/vehicle group than in the saline/vehicle group (Figure 3E). The anti-IL-5 antibody at any dose did not reduce the grade of vascular lesions compared to that observed in the 1% OVA/vehicle group (Figure 3E).

### 3.2 Effects of anti-IL-5 antibody on blood cytokine levels in eosinophilic vasculitis mouse model

**FIGURE 4:**
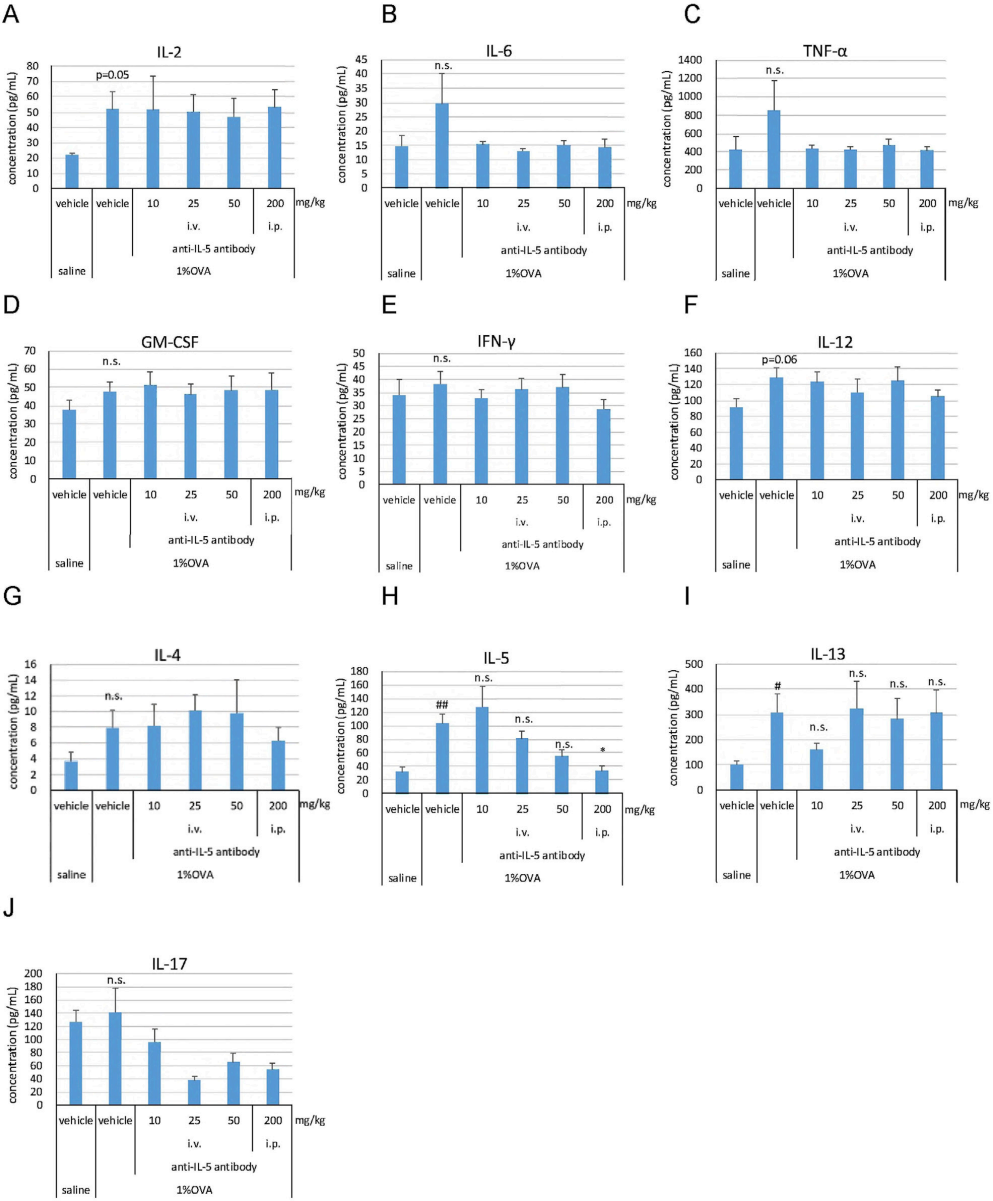
Effects of the anti-IL-5 antibody on blood cytokine levels in the mouse eosinophilic vasculitis model. Blood was drawn from the tail on the third day of inhalation, and plasma was obtained to measure IL-2 **(A)**, IL-6 **(B)**, TNF-α **(C)**, GM-CSF **(D)**, IFN-γ **(E)**, IL-12 **(F)**, IL-4 **(G)**, IL-5 **(H)**, IL-13 **(I)**, and IL-17 **(J)**. i.v., intravenous; i.p., intraperitoneal. Data are expressed as the mean +SEM, n = 5–7. #*P* < 0.05, ##*P* < 0.01 vs. saline/vehicle group (Student's *t*-test), *P < 0.05 vs. 1%OVA/vehicle group (Dunnett’s multiple comparison test). n.s., not significant.

Several cytokines reported to be involved in the pathogenesis of EGPA were analyzed in the blood. The production of T-helper (Th) 2-related cytokines, IL-5 and IL-13, was significantly higher (*P* < 0.01 for IL-5, *P* < 0.05 for IL-13) in the 1% OVA/vehicle group than that in the saline/vehicle group (Figures 4H, I). No significant changes were observed in the levels of IL-2, IL-6, tumor necrosis factor (TNF)-α, granulocyte-macrophage colony-stimulating factor (GM-CSF), interferon (IFN)-γ, IL-12, IL-4, and IL-17 in the 1% OVA/vehicle group compared to those in the saline/vehicle group (Figures 4A-J); however, some cytokines (IL-2 and IL-12) showed an increasing trend (*P* = 0.05 for IL-2, *P* = 0.06 for IL-12) (Figures 4A, F). The anti-IL-5 antibody (200 mg/kg, i.p.) significantly suppressed the production of IL-5 (*P* < 0.05) (Figure 4H); however, it did not suppress the production of IL-13 (Figure 4I).

## 4 Discussion

The aim of this study was to evaluate and characterize the usefulness of an OVA-induced eosinophilic vasculitis mouse model in elucidating the effects of an anti-IL-5 antibody. The results showed that the anti-IL-5 antibody did not sufficiently suppress the development of vasculitis in this model.

The previously developed mouse model of OVA-induced eosinophilic vasculitis (Yamauchi et al., 2010) was used for this study. The findings observed with the mouse model, i.e., the eosinophil counts in the blood and BALF and lymphocyte counts in BALF, the vascular lesion grade, and the levels of certain cytokines in the blood (i.e., IL-4, IL-5, and IFN-γ), were similar to those reported by Yamauchi et al. Therefore, we conclude that the animal model established herein aligns with the previously reported model.

In this study, we used reslizumab instead of mepolizumab, a drug approved in many countries, including the United States by the Food and Drug Administration (FDA), for the treatment of EGPA. According to the FDA’s Pharmacology review for mepolizumab (FDA, 2015), mouse and human IL-5 exhibit approximately 70% homology; however, mepolizumab cannot bind with high affinity to mouse IL-5. In contrast, according to the European Medicines Agency (EMA) Public Assessment Report, reslizumab can bind and inhibit human and mouse IL-5 with similar affinities (EMA, 2016). Mepolizumab and reslizumab are reported to both bind to the IL-5 epitope within the IL-5Rα-binding domain with similar affinity (Amini-Vaughan et al., 2012). In a recent pilot study of reslizumab, efficacy and safety were demonstrated in patients with EGPA (Manka et al., 2021). For these reasons, reslizumab was used in this study. The dosage of reslizumab used in this study was determined based on previously reported information (FDA, 2016) and the maximum dose that could be administered to mice based on the formulation specifications.

Our findings indicated that the anti-IL-5 antibody clearly affected the eosinophil counts in the blood and BALF but did not reduce the lymphocyte counts in the BALF, compared to that of the 1% OVA/vehicle group. This was consistent with the report indicating that anti-IL-5 antibodies reduced BALF eosinophil counts but did not reduce BALF lymphocyte counts in a mouse model of OVA-induced airway-hyperresponsiveness (Hamelmann and Gelfand, 1999). In the clinical setting, mepolizumab reduces blood eosinophil counts in patients with severe eosinophilic asthma (Büttner et al., 2003). Thus, IL-5 is known to primarily regulate eosinophils, and anti-IL-5 antibodies are unlikely to exert a direct effect on lymphocyte infiltration.

In addition, the present study demonstrated that the anti-IL-5 antibody did not suppress the formation of vascular lesions compared to the effect observed in the 1% OVA/vehicle group. These results indicate that factors other than eosinophils are likely to be important in the formation of vascular lesions in this mouse model. Previous studies using the same mouse model have reported that several drugs partially reduce eosinophil counts in BALF, and consequently ameliorate vascular lesions (Suzuki et al., 2013; Koizumi et al., 2014; Oikawa et al., 2016; Matsumoto et al., 2019). However, given that these drugs have reduced the production of several cytokines and the number of intraluminal myofibroblasts, it is plausible that the observed suppression in vascular lesions is mediated by a mechanism independent of eosinophils. Anti-IL-5 antibody did not suppress BALF lymphocyte counts in the present study, suggesting that lymphocytes, rather than eosinophils, may be a crucial factor in the development of vascular lesions.

To further determine the effect of the anti-IL-5 antibody on lymphocytes contributing to pathogenesis, cytokine concentrations in the blood were measured exploratorily. In a previous report (Yamauchi et al., 2010), cytokine production was higher on the third day of aerosol exposure than on the seventh day in both the blood and BALF in the eosinophilic vasculitis mouse model. Therefore, the blood cytokine levels were measured on the third day after aerosol exposure. In the 1% OVA/vehicle group, the production of IL-5 and IL-13 was significantly increased, whereas that of IL-2, IL-6, TNF-α, GM-CSF, IFN-γ, IL-12, IL-4, and IL-17 did not show significant changes compared to that in the saline/vehicle group. The cytokine profile of IFN-γ, IL-4, and IL-5 was mostly consistent with that of Yamauchi’s model (Yamauchi et al., 2010). The increased lymphocyte infiltration in lung tissue, coupled with elevated blood levels of IL-5 and IL-13, suggests a contribution of Th2 cells to disease pathogenesis. The anti-IL-5 antibody demonstrated a dose-dependent suppression of IL-5 production. However, it did not exhibit any impact on the production of other cytokines, or the effects were inconclusive, suggesting that it did not directly affect the production of cytokines other than its direct target (i.e., IL-5). This does not contradict the fact that mepolizumab does not reduce T-cell function in patients with severe eosinophilic asthma (Büttner et al., 2003). Therefore, we conclude that there is essentially no significant difference in IL-5 function between mice and humans. Increased infiltration of eosinophils and lymphocytes as well as the production of cytokines, including Th1, Th2, and Th17, are known to be involved in the pathogenesis of EGPA (Vaglio et al., 2013). Therefore, further investigation (e.g., flow cytometry analysis) is needed to elucidate the types of lymphocytes, especially the Th cells, involved in disease pathogenesis in this mouse model.

The involvement of non-eosinophilic cells, especially Th and B cells, has been suggested in the pathophysiology of EGPA (Vaglio et al., 2013). Non-eosinophilic inflammation in patients with EGPA has been hypothesized to potentially contribute to the lack of response to mepolizumab treatment (Wechsler et al., 2017). Therefore, it is conceivable that eosinophilic and/or non-eosinophilic pathogenesis may be involved depending on the patient. In clinical settings, the efficacy of rituximab, an anti-CD20 antibody, for EGPA has also been reported (Akiyama et al., 2021). In addition to Th cells and B cells, Kotas et al. (2021) recently reported a model of eosinophilic vasculitis and pulmonary hemorrhage induced by intranasal IL-33 in hypereosinophilic mice (i.e., IL-5-transgenic mice). Their model recapitulated aspects of eosinophilic vasculitis in patients with EGPA, in which type 2 innate lymphoid cells and Th2 cells play a major role in the development of eosinophilic vasculitis. However, in their analysis, the presence of hemorrhage was an indication of structural damage to the capillaries (Kotas et al., 2021); hence, the lesion of the arterial structure could not be determined directly through a pathohistological examination. They also characterized their model by performing a respiratory function assessment. Due to the complex nature of EGPA, characterized by the formation of lesions throughout the body, it is important to recognize the limitations of directly applying these findings to clinical settings. It is also necessary to consider the differences in responsiveness due to variations in the immune systems between mice and humans. For example, while IL-5 is active on B cells in mice, this activity is not observed in humans (Takatsu, 2011). Further studies are required to elucidate the underlying mechanisms and characterize the usefulness of the OVA-induced eosinophilic vasculitis mouse model. This may include evaluating the effect of anti-IL-5 antibodies on respiratory function and investigating the effects of other agents (e.g., anti-IL-13 and anti-IL-33 antibodies, as well as their combination with anti-IL-5 antibodies). To verify the involvement of B cells in this model, investigating the effects of B-cell depletion (e.g., using an anti-CD20 antibody) is of particular interest.

The non-clinical data from the present study indicate that IL-5 signaling is not primarily involved in the development of vasculitis in the OVA-induced eosinophilic vasculitis mouse model. These findings indicate that lymphocytes and/or cytokine signals other than or in addition to IL-5 are involved in the pathogenesis of this mouse model. This model could partly represent the pathophysiological features observed in patients with both eosinophilic and/or non-eosinophilic inflammatory responses, and may be useful for drug discovery in treating EGPA.

## Data Availability

The original contributions presented in this study are included in the article. Further inquiries can be directed to the corresponding author.
